# On Puerperal Convulsions

**Published:** 1808-12

**Authors:** 


### *To the Editors of the Medical and Physical Journal.*

Gentlemen,

In the vast and gloomy catalogue of diseases, to which the human frame is liable, there is none so replete with terrors, so inconceivably dreadful as that of Puerperal Convulsions; and none that requires nicer management and skill, and more boldness and promptitude in practice. I allude to those cases which occur after delivery, or during the time of actual labour.

That this disease depends upon pressure of the brain from extravasation, or from over-distention of its vessels, producing inflammation, and not from *nervous irritation*, is, I believe, a truth, that requires more generally to be understood.

It is no difficult matter to distinguish a disease from its symptoms when it exists, but it is a knowledge of the causes that must dictate a proper and effectual mode of treatment. Epilepsy is a disease we are all of us familiar with, but we know nothing of its causes, and, consequently, as little of its cure. This, certainly, then, can be an object of no small moment, as it may frequently, if not always, enable us, by timely care and proper management, to arrest a disease in its progress, which, when it occurs, is so fatal in its consequences.

The two following cases, which have lately come under my inspection, may serve to throw some light upon this important subject.

### CASE I.

A young woman, aged 19, had a difficult though natural labour of her first child; the pains were violent and protracted, tracted, which continued for eight or ten hours, when she was safely delivered by a respectable midwife. In the latter stage of the labour, she complained of pain and giddiness in the head; and as soon as the placenta was expelled, she sunk into a comatose state; and in ten minutes was seized with convulsions. She had three violent paroxysms, succeeded by slight intermissions; and in five minutes after the last, she died with every symptom of apoplexy. In this case, nothing was done, as I only arrived in time to see her expire; which was in about half an hour after she was delivered.

### CASE II.

A woman, advanced in years, was safely delivered of her second child, which was many years subsequent to her former. She was in labour for twelve hours, the latter part of which was unusually difficult. She appeared tolerably well, but complained of pain in the head, which gradually increased, and she became delirious; in which state she continued for two hours, and was seized with convulsions. Before I saw her she had had thirteen fits in rapid succession, which left her in a state of complete torpor, and she was to all appearance on the brink of dissolution. Her pulse was scarcely perceptible, and her skin was of a dark or purple colour. From these symptoms, I entertained no hopes of recovery, but ordered her a stimulant draught with æther and laudanum, &c. which seemed to revive her, and a second was given; when her natural colour was gradually restored, and the convulsions returned with the same violence as before. Her pulse now became strong and hard: and a considerable fullness appearing about the head, I took away a pint of blood from the arm, and applied four leeches to each temple: after which, her head was shaved, and a blister applied over the whole. Her bowels being in a costive state, a solution of soft soap was ordered as a glister, and an opening mixture with neutral salts and infus. sennæ. The bleeding having reduced her almost to a state of syncope, she sunk into a comatose sleep, in which she continued for six hours, when she awoke and was for a few minutes quite sensible, but again became delirious, and uttered the most incoherent expressions. As her pulse did not warrant a second bleeding, and the convulsions having entirely subsided, she took draughts every three hours with mixtur. camph. aq. ammon. acet. et vin. antim. which produced a most copious and general perspiration; in which treatment

